# Default Pathway of *var2csa* Switching and Translational Repression in *Plasmodium falciparum*

**DOI:** 10.1371/journal.pone.0001982

**Published:** 2008-04-23

**Authors:** Bobo W. Mok, Ulf Ribacke, Niloofar Rasti, Fred Kironde, Qijun Chen, Peter Nilsson, Mats Wahlgren

**Affiliations:** 1 Department of Microbiology, Tumor and Cell Biology (MTC), Karolinska Institutet, Stockholm, Sweden; 2 Swedish Institute for Infectious Disease Control (SMI), Stockholm, Sweden; 3 Department of Biochemistry, Faculty of Medicine, Makerere University, Kampala, Uganda; 4 Department of Gene Technology, School of Biotechnology, Royal Institute of Technology, Stockholm, Sweden; University of California Los Angeles, United States of America

## Abstract

Antigenic variation is a subtle process of fundamental importance to the survival of a microbial pathogen. In *Plasmodium falciparum* malaria, PfEMP1 is the major variable antigen and adhesin expressed at the surface of the infected erythrocyte, which is encoded for by members of a family of 60 *var-*genes. Peri-nuclear repositioning and epigenetic mechanisms control their mono-allelic expression. The switching of PfEMP1 depends in part on variable transition rates and short-lived immune responses to shared minor epitopes. Here we show *var*-genes to switch to a common gene that is highly transcribed, but sparsely translated into PfEMP1 and not expressed at the erythrocyte surface. Highly clonal and adhesive *P. falciparum*, which expressed distinct *var*-genes and the corresponding PfEMP1s at onset, were propagated without enrichment or panning. The parasites successively and spontaneously switched to transcribe a shared *var*-gene (*var2csa*) matched by the loss of PfEMP1 surface expression and host cell-binding. The *var2csa* gene repositioned in the peri-nuclear area upon activation, away from the telomeric clusters and heterochromatin to transcribe spliced, full-length RNA. Despite abundant transcripts, the level of intracellular PfEMP1 was low suggesting post-transcriptional mechanisms to partake in protein expression. *In vivo*, off-switching and translational repression may constitute one pathway, among others, coordinating PfEMP1 expression.

## Introduction

Pathogens constrained to survive within a mammalian host are under evolutionary pressure to acquire mechanisms that favor a chronic infection. Asexual *Plasmodium falciparum* endure by means of antigenic variation. Manifestations of this success are the recrudescence of parasites, the occurrence of super-infections, the establishment of chronic asymptomatic infections, and the paucity of sterile immunity. The maintenance of antigen expression is coordinated by the spleen, given that parasites of splenectomized human and animal hosts do not sequester and do not express PfEMP1 on the infected erythrocyte surface [1]–[3]. How *P. falciparum* manages to coordinate the expression of its variable antigens to subsist in the host is intriguing and in part unexplained.

Immune evasion of *Plasmodium falciparum* infected erythrocytes (IE) is predominately mediated by the antigenically variable and highly polymorphic cell surface antigen *Plasmodium falciparum* erythrocyte membrane protein 1 (PfEMP1). PfEMP1 is a major adhesin that mediates binding to a variety of host-receptors causing sequestration of IEs in the microvasculature of various organs and severe disease in children and pregnant women. Pregnancy-associated malaria (PAM) constitutes one of the malaria syndromes where the involved PfEMP1 species and host receptors have been characterized. VAR2CSA, which is also of particular interest for the present study, has previously been identified as the major PfEMP1 implicated in PAM and shown to interact with chondroitin sulphate A (CSA), non-immune immunoglobulins and possibly other host-receptors, engendering placental sequestration and pathogenesis in PAM [4]–[9]. Understanding the mechanisms that regulate the expression of a particular PfEMP1 is an important piece of the puzzle of understanding disease pathogenesis and may help to identify additional targets for combating the disease. Yet, how the parasite switches on and off PfEMP1 expression is at present only partly understood.

PfEMP1 is encoded for by members of the multi-gene family *var*. Each parasite genome harbors approximately 60 *var*-genes of high sequence diversity [10] but at any given time, only a single PfEMP1 species is expressed in each parasite. It has been demonstrated that this mutually exclusive expression of PfEMP1 is regulated at the level of *var*-gene transcription initiation [11]–[13] unlike mammalian systems where regulation occurs through negative feedback at the level of protein production [14]. Epigenetic mechanisms involving chromatin modification, activation or silencing by sterile genetic elements and repositioning of *var* loci in sub-nuclear compartments also play important roles in the switching and exclusive expression pattern of *var*-genes [15]–[21]. Further, the parasite carries an epigenetic memory at the level of the chromatin involving the methylation of the histone H3 [22].

It has until recently been controversial whether a single full-length *var*-gene is being exclusively transcribed or multiple *var*-genes are simultaneously present [23]–[25]. Using microarrays specifically designed to detect *var*-genes, with multiple probes covering the entire length of every *var*-gene in the genome, it was recently found that several full-length *var*-genes are being transcribed simultaneously but also that short spurious transcripts are present [26]. The untranslated full-length *var*-genes in fact share the same group of upstream promoter sequences as the translated, implying that “loose” transcription [12], [13] might be attributable to cross *var-*gene transcriptional activation within the same group [26]. Still there is one *var* transcript that is dominant both in ring- and trophozoite stages which is later translated into the corresponding PfEMP1 [23], [25].

The expression dynamics of *var*-genes and their switching rates have previously been studied using different approaches including *in vitro* and *in vivo* assays and mathematical modeling [27]–[30]. To gain a better understanding of the succession of *var* switching we have here monitored two highly clonal parasites during *in vitro* growth without enrichment or panning for ≈200 generations (>1 year). The *var*-genes were studied using a comprehensive set of tools to monitor *var-* RNA and DNA (microarray, qRT-PCR, northern blot, fluorescent *in situ* hybridization (FISH)) and to follow the expression of PfEMP1 (cell-assays, immunoblotting, FACS). The results of the study suggest that *var*-gene switching, at least for a subset of genes, involves a programmed process where a large majority of parasites switch to transcribe a single member of the *var*-gene family, PFL0030c or *var2csa*, which however seems to be translationally repressed. The implications of our findings for the pathogenesis of disease and survival of the parasite *in vivo* are discussed.

## Results

### Var-gene expression and phenotypic changes in separate 3D7 clones over 200 generations

**Figure 1:**
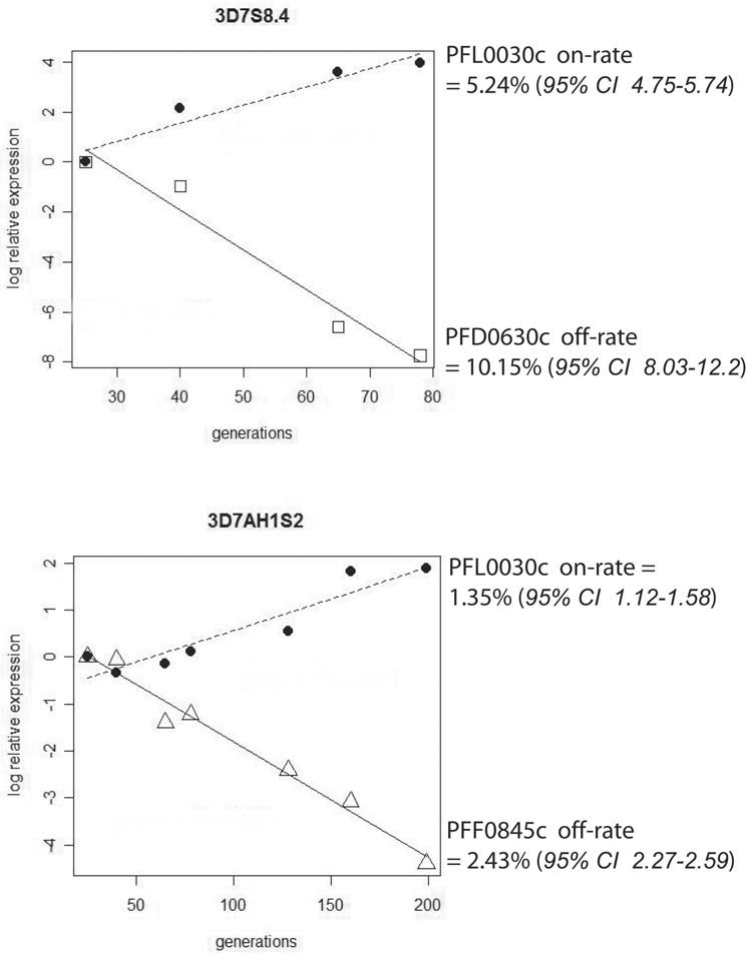
“On” and “Off” switching rate of dominant *var* and *var2csa* in 3D7S8.4 and 3D7AH1S2. Regression models were created by using the log of comparative expression level, log(2^ΔΔCt^) versus generations. In the case of 3D7S8.4, a linear range of regression was observed up to ∼100 generations. Thus, the *var* on/off rates for 3D7S8.4 were determined up to 100 generations, whilst for the 3D7AH1S2, the estimations were made up to 200 generations, as the linear regression was maintained for a longer time period. The *var* on/off rates for 3D7S8.4 and 3D7AH1S2 were determined by the 2^−ΔΔCt^ method, where ΔΔCt = (Ct_var_−Ct_house-keeping_)*t_n_*−(Ct_var_−Ct_house-keeping_)*t_0_*. The value for the first data collection (*t_0_*) was set as the baseline level (i.e. 15 and 25 generations for 3D7S8.4 and 3D7AH1S2, respectively.) The fold-change in the transcription compared to the baseline level was plotted, and the on/off rates of particular *var-*genes were estimated by the exponential function of the slope of the regression.

**Figure 2:**
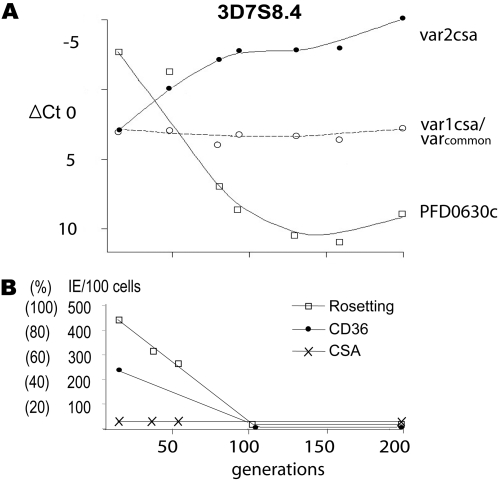
Changes in *var*-gene expression and adhesive phenotypes in clonal *P. falciparum* 3D7S8.4 over 200 generations of *in-vitro* growth. The transcription levels were determined by qPCR and are presented as ΔCt values being the difference in cycle threshold between the *var*-gene and the *seryl-tRNA synthetase* housekeeping gene (control). The transcription levels of *var_common_* and of *var*-genes that were dominantly expressed at particular time points are shown. The adhesive phenotypes of the IE of the two parasites were followed for rosetting, CSA- and CD36-binding as described in Table 1 and the methods section.

To follow *var*-gene switching over time, two parasite clones were monitored for ≈200 generations during which, both RNA expression and phenotypic changes were investigated at least once a month. The parasites were 3D7S8.4 and 3D7AH1S2, which had been serially selected for either rosette formation or adhesion to CD36 and then cloned by micromanipulation. The rosetting clone 3D7S8.4 transcribed the *var*-gene PFD0630c at onset whilst the 3D7AH1S2 clone selected for CD36 adhesion transcribed PFF0845c. Both *var*-genes are internally located on chromosomes 4 and 6, respectively. The global transcriptional profiles of these early generation clones have previously been established during multiple array experiments and the results have been confirmed using northern blot and RT-PCR [26]. When following the RNA expression with qPCR over time we found that the transcription of both initially identified *var*-genes (PFD0630c/PFF0845c) were gradually switched off, with an off-rate of 10.15 and 2.43% per generation, respectively. A total loss of PFD0630c transcription was observed after 80–130 generations in 3D7S8.4, while it took 175–200 generations for 3D7AH1S2 to completely loose transcription of PFF0845c. Instead, transcripts of *var2csa* (PFL0030c) began to appear in both clones. The loss of the dominant *var*-genes was intimately linked to an increase of *var2csa* (PFL0030c) transcription both in 3D7S8.4 and 3D7AH1S2, with on-rates of 5.24% and 1.35% per generation, respectively (Figure 1 & 2).

Down-regulation of the initial *var* transcription was strictly paralleled by off-switching of corresponding adhesive phenotypes. Rosetting and CD36 binding began to decline already after 25 generations of growth and had disappeared at 80–100 generations in 3D7S8.4, and no CSA binding was detected in this parasite (Figure 2). CD36 binding was one third of the original level in 3D7AH1S2 after 100 generations of growth and was not seen in the late generation parasites (Table 1 and data not shown).

**Table 1:** Adhesion profile of 3D7AH1S2 and 3D7S8.4 at early and late generations

Clones Generations	3D7S8.4		3D7AH1S2	
	16	>190	16	>190
Rosetting	≈90%	-	≈10%	-
Autoagglutination	-	-	-	-
CHO-CD36^a^	≤250	≤10	≈500	≤10
CHO-ICAM^a^	≤50	≤10	≤50	≤10
CHO^a^	≤50	≤10	≤50	≤10
Soluble Heparin^b^	-	-	-	-
Soluble CD31^b^	-	-	-	-
CSA^cd^	-	-	-	-
TSP^c^	-	-	≈175	-
Placenta^e^	≈500	-	-	-

a: Number of IEs bound per 100 cells; b: % of IEs showing surface fluorescence when incubated with Alexa-labelled antigens; c: CSA or thrombospondin (TSP) -coated plastic (50 µg/ml); d: A postive control parasite FCR3CSA bound at ≈235±45 IEs/mm^2^; e: Number of IEs bound to 1 mm^2^ of placental tissue; -: Lack of detectable adhesion

**Figure 3:**
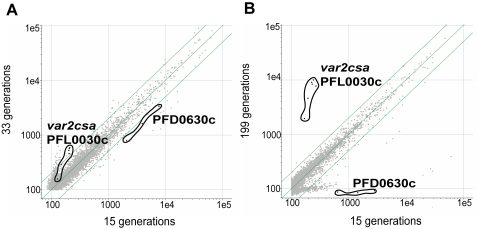
Comparisons of global transcriptomes of 3D7S8.4 as studied by microarray analysis. RNA was obtained from 15 vs. 33 generations and 15 vs. 199 generations. (A) off-switching of the originally dominant *var* PFD0630c and on-switching of PFL0030c (*var2csa*) was already seen at 33 generations; (B) *var2csa* (PFL0030c) was highly up-regulated in long-term cultured parasites and almost no PFD0630c was detected (background signal level) at 199 generations.

Having found that both 3D7S8.4 and 3D7AH1S2 converge to *var2csa* transcription, matched by the loss the binding ability, we decided to explore the underlying mechanisms in some detail. 3D7S8.4, which rapidly lost its binding phenotype and had a faster off- and on- switching rate of the dominant *var-*genes, was therefore chosen for further analyses. To confirm the data generated by qRT-PCR we regularly followed the parasite using microarrays (Figure 3). As can be seen from Figure 3, the *var*-gene PFD0630c was dominantly transcribed in the 15 generation parasites (Fig. 3A), while expressed to a lesser extent in 33 generation parasites and completely absent in those grown for ≈200 generations. The *var2csa* gene appeared to be dominantly transcribed in the long-term cultured non-selected parasites. The oligonucleotide coverage of *var2csa* was high, with four detecting the DBL-1x, DBL-4ε, DBL-5ε domains and the upstream open reading frame (uORF; chr12_glimmerm_22). The remaining two oligonucleotides targeted the DBL-6ε domain at the 3′ end. Significantly higher signals from all six oligonucleotides were observed in the long-term cultured 3D7S8.4 parasites (199 generations) as compared to the recently cloned one (15 generations; Figure 3B), implying that the *var2csa* transcripts were full length from the 5′ to the 3′ end of the exon-I of the gene.

### Transcription of full-length *var2csa* mRNA in sub-clones

**Figure 4:**
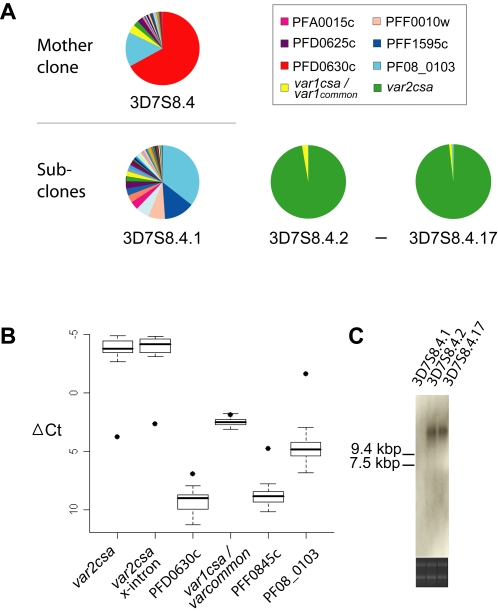
Expression of *var2csa* and other *var-*genes after long-term cultivation of *P. falciparum in vitro*. (A) The pie charts show the relative transcription level of each *var-*gene in the different clones. The original 3D7S8.4 clone (15 generations) dominantly expressed the PFD0630c, but sub-populations of other *var-*genes were also detected. In contrast, the *var2csa* gene was found to be highly expressed in 16 out of 17 sub-clones (3D7S8.4.2-3D7S8.4.17) but in one (3D7S8.4.1) generated from the long-term cultured ∼200 generations 3D7S8.4. (B) Expression levels of different *var*-genes in the 3D7S8.4 sub-clones, 3D7S8.4.1-17. *var2csa* was highly expressed in all the subclones except for 3D7S8.4.1 (•), which dominantly transcribed PF08_0103. *var_common_* was expressed in all the parasites though at a relatively low level. (C) Northern-blot analysis of *var2csa* expression in total RNA extracted from the sub-clones 3D7S8.4.1, 3D7S8.4.2 and 3D7S8.4.17. The membrane was hybridized with a *var2csa* DBL1-2x probe. Comparable amounts of total RNA from each sample were loaded as shown in the ethidium bromide-stained gel (lower panel).

To further investigate the expression of *var2csa* and other *var*-genes, 3D7S8.4 was sub-cloned at 200 generations by micromanipulation, thereby generating a set of 17 new clones (3D7S8.4.1 to 3D7S8.4.17). Transcription of all clones was investigated using a set of qPCR primers covering all *var*-genes (Figure 4A & B). 16 out of 17 clones showed identical profiles with high levels of *var2csa* transcription. An exception was clone 3D7S8.4.1, which dominantly expressed another *var*-gene (PF08_0103). The pseudogene *var1csa*/*var*_common_ (PFE1640w) was constitutively expressed in all parasite samples, though at a fairly low level (Figure 4B), in line with previous observations on the ubiquitous nature of constitutive transcription of this *var* species across parasite isolates [31], [32].

To investigate whether the *var2csa* expressed in the sub-clones was a correctly spliced transcript, we examined the RNA using qRT-PCR with primers mapping to opposite sides of the intron region (*var2csa* x-intron; Figure 4A). No difference in amplification was observed between the *var2csa* exon specific primers and the *var2csa* x-intron primers suggesting the presence of a spliced transcript. Northern blot was further used to verify the full-length feature of the *var2csa* transcript. Abundant *var2csa* transcripts of approximately 13 kb were present in the sub-clones, a molecular size corresponding to that of *var*-gene mRNA in CSA selected parasites (Figure 4C and not shown). We thus conclude that the samples do not contain any DNA contaminants or unspliced transcripts implying functionality of the *var2csa* mRNA detected.

### Sparse PfEMP1 production and lack of PfEMP1 surface expression albeit high level of *var2csa* transcripts

No specific binding of the IE of long-term cultured parasites (≈200 generations) was seen to either cells or different host receptors albeit the high level of *var2csa* transcription detected. Rosetting was absent as was binding to CD31, CD36, ICAM-1, HS, CSA, TSP, CHO-cells and placental tissue (Table 1).

**Figure 5:**
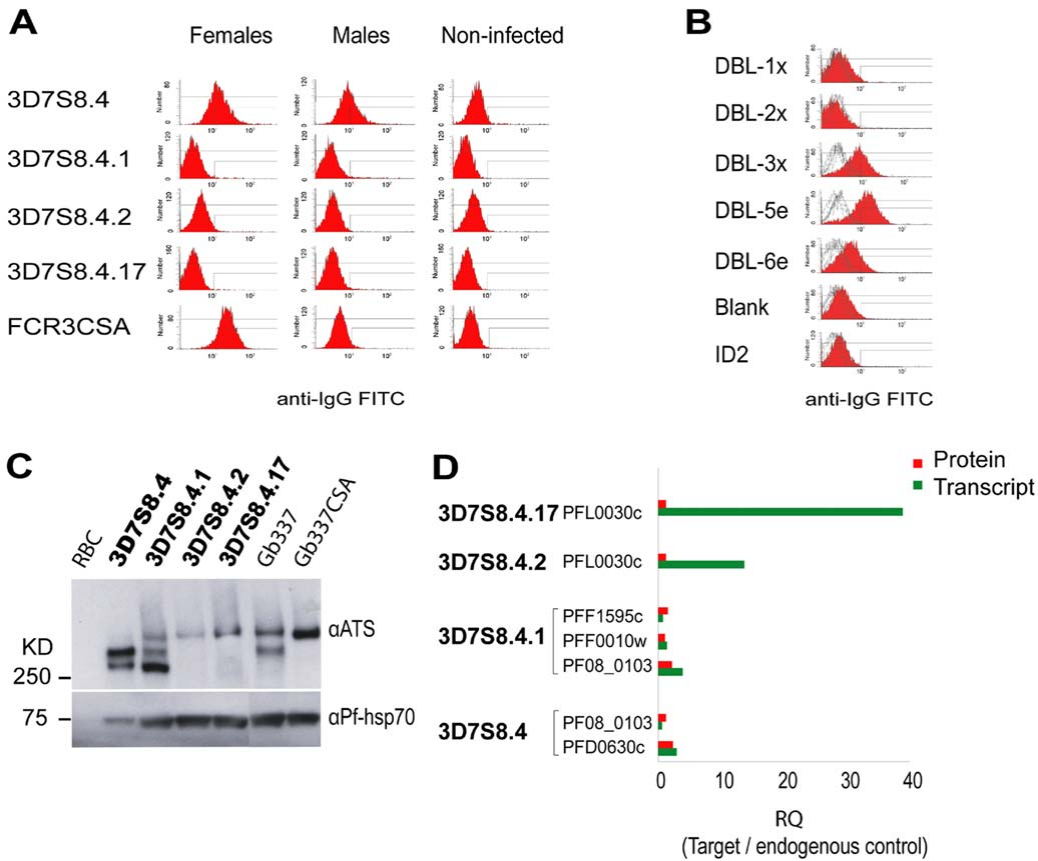
Detection of PfEMP1 in different *P. falciparum* clones by flow cytometry- and immunoblotting. The IEs were labeled (A) with IgG from plasma pools of *P. falciparum* exposed males (Males), females (Females) or non-infected Danes, and (B) IEs used in (A) were labeled with IgG from rabbit-sera raised against different domains of VAR2CSA (DBL-1x; DBL-2x, DBL-3x, DBL-5e, DBL-6e) or with a serum raised to an irrelevant control antigen (ID2), positive control (FCR3CSA) is shown in red. (C) The blot illustrates the PfEMP1 expression in whole cell-lysates of late trophozoite infected RBCs of the 3D7S8.4 early and late generation clones. Parasites with bolded lettering are described in greater detail in D. The blot was incubated with a PfEMP1 antibody raised against the conserved part of the acidic terminal segment (ATS). Anti-PfHsp-70 reactivity was used as loading control. Uninfected RBCs were included as negative control. Gb337 and Gb337CSA are included as positive controls of VAR2CSA expressing parasites. Whilst Gb337 is heterogeneous in its PfEMP1 expression, upon repeated panning on CSA, only the higher molecular weight protein is expressed. D) The graph illustrates the inter-relationship of the transcript versus protein levels of the dominant *var*-gene(s) in the 3D7S8.4 early and late generation clones.

To confirm the lack of VAR2CSA surface expression, IEs were further studied by flow cytometry using different sera and IgG preparations. IgG specific for DBL1x, DBL2x, DBL3x, DBL5e and DBL6e of VAR2CSA raised in rabbits as well as pooled plasma from *P. falciparum*-exposed men or multi-gravid women from Ghana were used. The IEs of the original rosetting clone 3D7S8.4 studied at 15 generations was recognized to a similar degree by the plasma pools from men and pregnant women, but not by the different anti-VAR2CSA IgG. However, the non-adhesive IEs of 3D7S8.4 grown for ≈200 generations *in vitro* was neither recognized by the pooled patient sera of either sex, nor by the anti-VAR2CSA IgGs. The same was true for IEs of the sub-clones 3D7S8.4.1, 3D7S8.4.2 and 3D7S8.4.17 (Figure 5A & B). In contrast, IE of the control parasite FCR3CSA and Gb337CSA that had been repeatedly selected for adhesion to CSA showed good differential reactivity with the plasma pool obtained from pregnant women versus those of men and was well-recognized by IgG raised to the DBL3x, the DBL5e and the DBL6e domain of VAR2CSA (Figure 5A & B and data not shown).

Having found that there was no serum- or IgG reactivity with the IE surface of the long-term cultivated parasites, we subsequently performed immunoblot analysis to investigate whether the *var2csa* transcripts were translated into protein, using an anti-PfEMP1 monoclonal antibody preparation (anti-ATS). In the mother clone 3D7S8.4, a ∼290 kDa band was dominantly labeled, corresponding well to the size of the major *var* transcript identified in this parasite (PFD0630c); in the control parasite Gb337CSA it appeared as a band of ∼340–360 kDa whilst in the switch clone 3D7S8.4.1 which dominantly transcribed *var*-gene PF08_0103 it appeared as a ∼250 kDa band. In concordance with the estimated size of the dominant *var2csa* transcript (∼355 kDa), a faint high molecular weight band was also observed in the long-term cultured sub-clones 3D7S8.4.2 and 3D7S8.4.17. However, despite the high level of *var2csa* transcripts in these parasites, the relative abundance of the translational products was sparse, hence contrasting the inter-relationship of transcript versus protein levels of the major PfEMP1s in the non-*var2csa* expressing clones (3D7S8.4 & 3D7S8.4.1) (Figure 5 C & D). This finding implies that the translational products of *var2csa* in the long-term cultured parasites are significantly controlled through post-transcriptional mechanisms [33].

### Activation and silencing of the *var2csa* gene involve sub-nuclear repositioning

**Figure 6:**
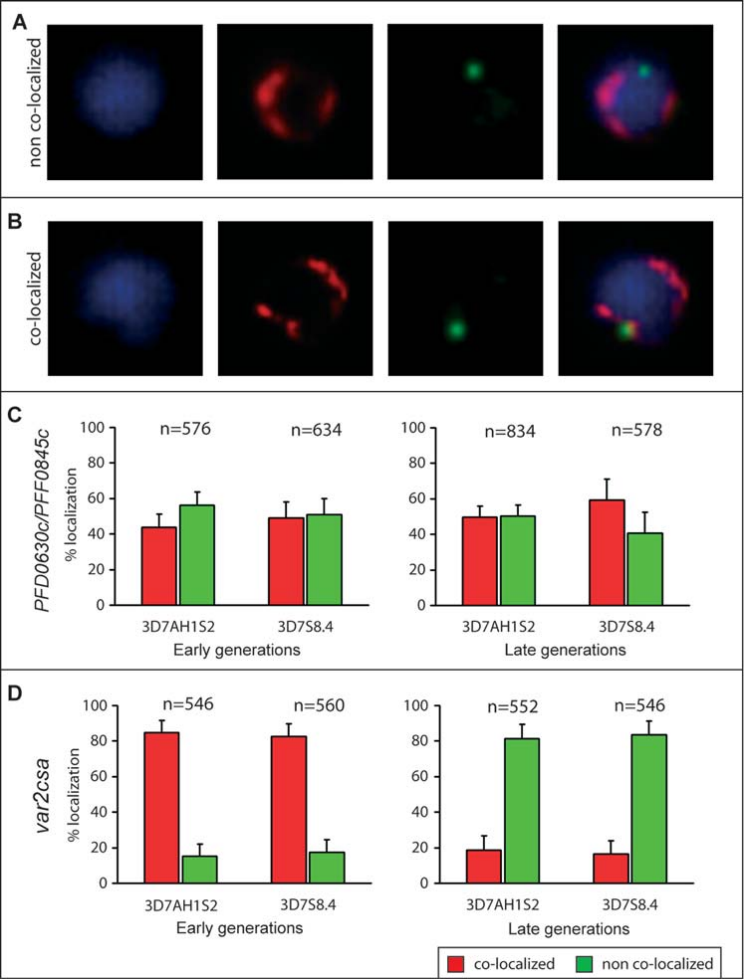
Repositioning of *var*-genes in the peri-nuclear area as seen by FISH in *P. falciparum* 3D7S8.4 and 3D7AH1S2 ring-stage parasites at onset and after 200 generations of *in vitro* growth. (A & B) Representative pictures illustrate different localization patterns of telomeric clusters (rep20, red probe) compared with that of a *var*-gene (green probe) in nuclei stained with DAPI (blue). Telomeric clusters appear as horseshoe-like bundles where condensed heterochromatins are located. (C) The *var*-genes expressed at the onset of the experiment (PFD0630c/PFF0845c) did not reposition and did not show any correlation between their transcriptional states and peri-nuclear positions in the same parasites. (D) In contrast, in early generation parasites (15–21 generations) *var2csa* is in a silenced state and co-localizes with the telomeric clusters. After 194-206 generations *var2csa* transcription is active in both parasites (3D7SS8.4, 3D7AH1S2) with a concordant repositioning from telomeric clusters to a zone without condensed genetic material.

Repositioning of *var* loci within the sub-nucleus has been found associated with the expression of subtelomerically located *var*-genes [17], [19]. We consequently studied the *var-*genes expressed in the cloned parasites (PFD0630c; PFF0845c) and the location of the telomere-repeats (rep20) using two-color fluorescent *in situ* hybridization (FISH; Figure 6). The great majority (>90%) of PFD0630c and PFF0845c were located at the rim of the nuclei (referred to as zone A by Ralph et al. [19]). Neither of the genes, which were of the upsC type and located internally on chromosomes 4 and 6, did however reposition in respect to telomere-repeats/heterochromatin as their transcriptional states changed. Their peri-nuclear gene locations (Figure 6C) were the same in parasites obtained immediately after cloning and in those obtained after long-term growth. Approximately half of the genes were located within the telomeric clusters/heterochromatin and approximately half outside of it (∼600 nuclei /gene counted). In contrast the *var2csa* gene was found to be associated with the telomeric clusters (rep20) at onset of the experiment while after long-term cultivation (194–206 generations), the activated *var2csa* gene dissociated away from the telomeric clusters and heterochromatin in more than 80% of the parasites (Figure 6D). As for the upsC *var*-genes, *var2csa* was located at the rim of the nuclei.

### Switching to *var2csa* is independent of genomic rearrangement

Using the microarray we monitored the parasite for global transcriptional changes throughout the study. Some of the genes were found down-regulated (Table S1) upon long-term *in vitro* propagation. Still, the skeleton-binding protein 1 (SBP1), previously shown to be required for PfEMP1 trafficking [34], was found transcribed at similar levels throughout the experiment and normal levels of RNA were present for genes encoding KAHRP and PfEMP3 when the parasites at early generations switched to transcribe *var2csa* (Table S1, Figure S1) suggesting that the lack of binding of the IE and surface expression was not due to deficient PfEMP1 transport at least in the <50–80 generation parasites (Figure 1). However, the transcription of the knob-associated histidine rich protein (KAHRP) and PfEMP3 was found absent in the 199 generation parasites due to a possible late spontaneous deletion of the left arm of chromosome 2 ([35]). Complementary comparative genomic hybridizations between 3D7S8.4 of 19 and 56 generations revealed only four genes (PFC1000w, PFE0635c, PF08_0138, PF10_0229) to have undergone either deletion or sequence polymorphisms in the later generations (not shown). This suggests that switching to *var2csa* happened before any major genomic rearrangements occurred, involving genes encoding proteins such as KAHRP or PfEMP3.

## Discussion

If it were so that a microbe would express antigenic variants in an un-coordinated manner the host would shortly raise an immune response to each and every variant and clear the infection. Parasites have consequently developed means to handle this host challenge. *P falciparum,* for example, successfully evades immune recognition of PfEMP1 by combining allelic exclusion of the *var*-genes with switching, yet the exact mechanisms by which this occurs are not known. To increase our understanding on *var*-gene switching we performed a systematic analysis of the transcriptional profile of *var*-genes over time in phenotypically distinct 3D7 parasite clones. In the absence of selective pressure, 95% of the parasites derived from a single clone eventually switched to transcribe a single member of the *var*-gene family, PFL0030c or *var2csa.* However, albeit the high levels of *var2csa* transcripts, the relative abundance of translated product was sparse and no PfEMP1 surface expression was observed, implying that *var2csa* may be a target for post-transcriptional regulation.

Sequence analysis of the 3D7 genome has revealed the *var*-gene repertoire to fall into different classes depending on chromosomal location, the orientation of the gene as well as on the upstream flanking sequence, the latter known as upsA, -B, -C, -D and -E. The two *var*-genes expressed in our parasites at onset belonged to the chromosomal internal cluster upsC *var*-genes (PFD0630c and PFF0845c). A recent study demonstrated that different *var*-genes have different intrinsic switching rates and that *var*-genes in centromeric location have higher “on” rates and lower “off” rates [36]. We found that this phenomenon does not apply to all central *var*-genes, at least not in the case of PFD0630c. In the present study, off-switching in 3D7S8.4 parasites was observed already at 33 generations of growth (<10 weeks). Furthermore, although the same *var-*genes (PFF0845c/MAL6p1.252) and the same genotypic parasites (here 3D7AH1S2) were monitored in both studies, the “off” rates were considerably dissimilar. In the present study the parasites had switched from the dominant *var-*gene (PFF0845c) already at 48 generations (<14 weeks), whereas in a study by Frank *et al* [36] the expression of the same dominant *var*-gene remained unchanged up to 66 generations (19 weeks). These findings may indicate that the switching rate is not inherited on genomic level but is rather under epigenetic control.

**Figure 7:**
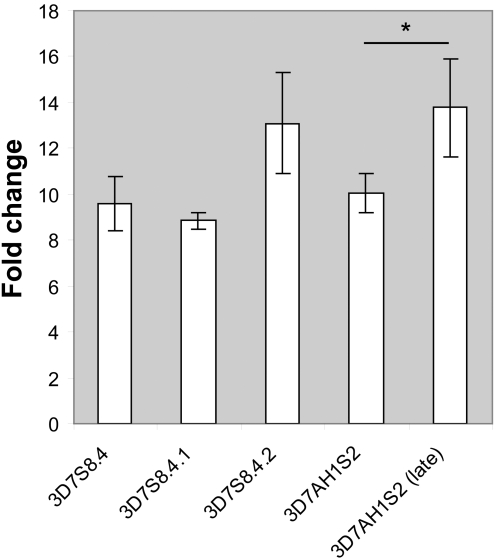
Quantitative differences in complete cycle multiplication rates between different parasites. Mean of fold change per erythrocytic cycle for at least three consecutive 48 hour cycles were calculated from blood smear parasitemias. Significant differences between “early” versus ”late” generation clones (3D7S8.4 vs 3D7S8.4.1; 3D7S8.4 vs 3D7S8.4.2 and 3D7AH1S2 vs 3D7AH1S2 late) were determined using an unpaired Student́s t-test at a 5% significance level. Analysis was performed in SPSS version 12.0. *P<0.05; Significantly higher multiplication rate was observed in the late S2-clone. The same trend was also observed in the other VAR2CSA expressing late generation clone S8.4.2, the difference here however was not statistically significant (P = 0.067).

An inter-relationship between the “off” and the “on” rates was found where “off“ rates of the original *var*-genes were about twice those of the *var2csa* “on” rates, arguing that switching to other intermediate *var*-genes also potentially occurred. Still, *var2csa* expression was dominant in the late generation parasites, which may in part be due to differences in proliferation rates in-between the *var2csa*- and non-*var2csa* expressing clones (Figure 7). It is possible that the higher multiplication rates in the *var2csa*-expressing clones endowed these parasites with a selective growth advantage over other switch variants. Further, we do not know whether the *var2csa* locus in the late generation parasites (3D7S8.4.2-17 sub-clones) would remain activated until an episode of exogenous challenge or whether they spontaneously would switch at a slow rate to another *var* variant. Upon panning of some of the sub-clones on placental sections or on CD36 expressing cells we could not retrieve any major binding IE populations (data not shown). This may suggest that this group of parasites does not revert back to PfEMP1 expression or that other types of stimuli may be required. It has to be noted that compared to other strains, such as HB3 and FCR3, it is difficult to retrieve 3D7 parasites panned on CSA [37]. On the other hand, up-regulation of *var2csa* transcription in 3D7 without selection is readily observed [36], [38].

As both parasite clones studied herein expressed upsC *var*-genes at onset and both eventually converged to *var2csa* transcription, and given that the *var* switching history of a particular parasite has been shown to result in a particular switching rate [30], it is possible that switching to *var2csa* is favored in parasites previously transcribing a particular group of *var*-genes (upsC). This, however, remains to be confirmed with a larger set of parasites both *ex vivo* and *in vitro*. Recent data suggest that transcription of in particular upsC *var*-genes predominate in children with asymptomatic infections [39]. Interestingly *var2csa* transcription, although mainly associated with pregnancy isolates, has occasionally been observed in samples from children with acute malaria [40], [41] and in peripheral-blood parasites of experimentally infected humans [24]. Moreover, performing a pilot study on other *in vitro* adapted parasites (FCR3, TM180 and 7G8), we found trace amounts of *var2csa* in FCR3 after 50 generations of growth without any selective pressure (data not shown).

Repositioning of *var* loci within the peri-nuclear area has been found to be associated with the expression of subtelomerically located *var*-genes. Similarly, the *var2csa* gene was here discovered to reposition from the telomeric clusters and the condensed heterochromatin to occupy a suggested site of transcription, supporting the notion that translocation of the *var2csa* gene into a suggested transcription site may in certain cases silence the transcription of other *var*-genes. However, although *var2csa* repositioned and was highly transcribed, the sparse presence of translational products and absence of surface expression imply an additional layer of control, which remains to be further investigated. The exploitation of transcriptional repression as a post-transcriptional regulatory mechanism has previously been described only for transcripts of *P. berghei* gametocyte origin [42].

**Figure 8:**
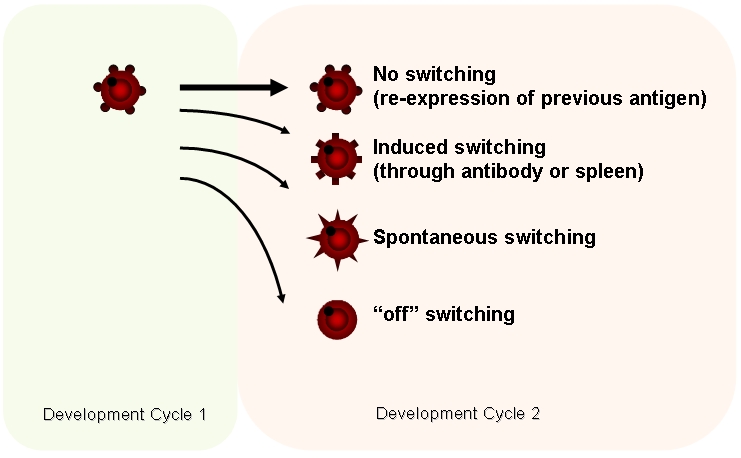
Suggested switching pathways of *var*-genes and their PfEMP1 in *P. falciparum.*

There is today good evidence to suggest that the *var2csa* gene encodes an important PfEMP1-species, a ligand associated with the sequestration of IEs in PAM [4]–[9]. This gene is however atypical to other *var*-genes and apart from having a different domain-architecture and an uncommon upstream flanking region it is remarkably conserved across different *plasmodium* species. A *var2csa* ortholog is for example present in *P. reichenowi*, a parasite which has been predicted to have diverged from *P. falciparum* several million years ago [43]. The expression of *var2csa* transcripts in isolates of children and adults during malaria infections and the obvious lack of antibody responses to the corresponding encoded protein in these groups of patients suggest the presence of *var2csa* off-switching *in vivo*. Yet, under what circumstances would a functional transcript avoid to produce a translational product, and is it compatible with parasite growth *in vivo* during an infection that an IE does not express a surface PfEMP1? One might speculate that *in vivo* a temporal expansion of parasites expressing a post-transcriptionally regulated gene may coordinate PfEMP1 expression by permitting splenic clearance of the off-switch IEs thus giving only the remaining minute populations of other variants a possibility to survive. Such a pathway, among others (see Figure 8), would protect against the rapid exhaustion of the variant-antigen repertoire.

It is possible that among the *var*-genes solely *var2csa* can be translationally repressed to avoid exposure of the corresponding PfEMP1 to the host immune system prior to pregnancy. The suppression may be released upon pregnancy by specific factors or conditions in the placenta allowing for expansion of parasites translating the PfEMP1var2CSA and expressing it at the IE surface. In the future, it will be interesting to study the prevalence of off-switching in different isolates including those obtained from splenectomized donors, and further assess what role post-transcriptional regulation has for different genes across the life cycle stages of *P.falciparum.*

## Materials and Methods

### Parasites and RNA preparation

The parasite 3D7AH1S2 was generated by multiple panning of 3D7AH1 parasites on CD36-CHO transfectants, followed by micro-manipulation cloning while 3D7S8.4 was selected twice from 3D7 parasites for the rosetting phenotype by micro-manipulation cloning. FCR3CSA was selected by repeated panning of FCR3 IE on CSA-coated Petri dishes [26]. 17 sub-clones (3D7S8.4.1-17) were randomly picked by micromanipulation from 3D7S8.4 after 200 generations of *in vitro* growth. Phenotypic characterization of the parasites was performed as described elsewhere [23] using trophozoite IE at 5–8% parasitemia. For RNA preparation, parasites were first tightly synchronized during three consecutive rounds of 5% D-sorbitol treatment. Parasites were then collected as trophozoite-IE (24±4 hours) and total RNA was isolated using Trizol reagent (Invitrogen, Carlsbad, CA) and stored at −80°C.

### Microarray

The *P. falciparum* genome microarray was used [26]. 20 µg of total RNA was labeled following an amino-allyl dye coupling protocol. Hybridization and washing were performed using a Lucidea automated slide processor (Amersham Biosciences) and slides were scanned with a GenePix 4000 B scanner (Axon Instruments). At least one dye-swap experiment per generation post-cloning was performed. Signals and local background intensities from each spot were retrieved using GenePix Pro 5.1 software (Axon Instruments). Spots that passed the quality controls (default settings), together with visual inspection, were analyzed using GeneSpring 6.1 (Silicon Genetics). Local background filtering was applied and LOWESS was adopted to normalize the data [44]. Protocols and microarray data are publicly available at www.ebi.ac.uk/arrayexpress: A-MEXP-75 and E-MEXP-1199.

### Quantitative real-time RT-PCR

Total RNA was treated with Deoxyribonuclease I (Ambion) to remove contaminating DNA. cDNA synthesis was performed with 1 µg of total RNA and reverse transcribed using Superscript III Reverse Transcriptase (Invitrogen) with random primers and OligoDT (Invitrogen) as described by the manufacturer. For qRT-PCR, we employed the primer set of Salanti et al. and conducted the experiment as previously described [7]. An additional primer pair targeting the flanking regions of *var2csa* (*var2csa* x-intron: forward 5′AATAATACCAGTGACATTCTGCAAAA3′ and reverse 5′ACACGTAAAAGGTCCACAGGTG 3′) was added for evaluation of splicing and DNA contamination. All experiments included primers for two housekeeping genes, *seryl-tRNA synthetase* and *fructose-bisphosphate aldolase*, as endogenous controls. Levels of *var* transcription were calculated by the ΔCt method, in which the Ct (cycle threshold) for each *var*-gene was compared with that for the endogenous controls.

### Northern Blot

3D7 *var2csa* (PFL0030c) was PCR amplified from 3D7 genomic DNA with primers PFL0030c/F (AAATGGAAATCCGAATGGG) and PFL0030c/R (TGAGTCAAGGGTGTGTTCTTGGGGGTAAACC) and then ligated into a T7 3′ element employing the TOPO-Tools system (Invitrogen) as recommended by the manufacturer. Digitonin-labeled anti-sense RNA was produced using the Dig RNA labeling kit (Roche). Total RNA (2 µg) from trophozoite stage parasites was transferred to nylon membranes (Roche). Blots were pre-hybridized in Dig Easy Hyb buffer and then probed with 100ng/ml Digitonin-labeled anti-sense RNA in Dig Easy Hyb buffer (Roche) at 65°C overnight. After hybridization membranes were washed twice with 2x SSC, 0.1% SDS at RT and twice with 0.5×SSC, 0.1% SDS at 65°C. Hybridized RNA was detected with the Dig Luminescent detection kit (Roche) as described by the supplier.

### SDS-PAGE and immunoblot analysis

Pigmented IEs were harvested using a MACS magnetic cell sorter (Miltenyi BioTec) and solubilized in reducing SDS sample buffer. The total parasite extracts were separated on 3–8% tris-acetate gradient gels (Invitrogen) and transferred to nitrocellulose membranes. Membranes were blocked in 5% non-fat milk powder and probed with a mouse monoclonal raised against the conserved C-terminal acidic-terminal-sequence (ATS) of PfEMP1s (1∶250) (a kind gift from A. Cowman). The membranes were stripped and re-probed with a mouse anti-Pfhsp70 (1∶2000) (a kind gift from C. Fernandez). The antibody was raised against the C-terminal part of hsp70 and differentially recognizes *P. falciparum* and not human Hsp70 [45]. Detection by enhanced chemiluminescence (ECL; Amersham Biosciences) was performed after secondary detection with sheep anti–mouse Ig-HRP conjugate (1∶5000, Amersham Biosciences).

### Flow cytometry

Variant surface antigen expression was tested by flow cytometry using plasma pools from *P. falciparum*-exposed women or men, or non-infected human serum. Specific VAR2CSA surface expression was assessed using antisera to VAR2CSA DBL1-3x, ID2 (inter-domain between DBL2x and DBL3x) and DBL5-6e. In brief, 2×10^5^ late-stage IEs were purified (to >75% parasitemia) by exposure to a strong magnetic field (Miltenyi BioTec), stained with ethidium bromide (Sigma) and sequentially incubated with 5 µl of plasma or antisera, 0.4 µl of goat anti-human IgG (Dako) and 4 µl of fluorescein isothiocyanate (FITC)-conjugated rabbit anti-goat IgG (Dako) in a total volume of 100 µl. Samples were washed 2 times in PBS with 2% fetal calf serum between each antibody incubation step as described elsewhere [46]. All plasma samples were tested simultaneously with each parasite isolate.

### Fluorescent in situ hybridization (FISH)

FISH was conducted according to previously described methodology with minor modifications [47]. The dsDNA probes targeting PFD0630c, PFF0845c and PFL0030c/*var2csa* were amplified using the specific primers PFD0630c/F (5′-GAT GAC GAC AAG CCA AAT ACC-3′), PFD0630c/R (5′-ACA TAA TCC GCC TCC AGT TC-3′), PFF0845c/F (5′-CGT TGG ATG ACT GAA TGG TCC G-3′), PFF0845c/R (5′-TCA CCG AGG TCT ATG CTG AAC TGG-3′), PFL0030c/F (5′-AAG GAT AGA ATG GAA TGG AAT GAG C-3′) and PFL0030c/R (5′-CAC CAA TCG TCA ACT TTT TCG G-3′). PCR products were cloned into pCRII-TOPO vector and transformed into One Shot TOP10 Chemically Competent Cells (Invitrogen) according to the supplier's recommendations. Plasmids containing *var*-gene fragments were labeled using the Fluorescein-High Prime and Rep20 containing pUC9 plasmids (a kind gift from A. Scherf) with Biotin-High Prime kit (Roche Applied Science). Highly synchronous parasites (18±2 hours post invasion) were isolated from their host erythrocytes using saponin (0.05% w/v) and deposited as monolayers on Poly-L-lysine coated microscope slides (Menzel-Gläser). Air-dried monolayers were fixed with 4% paraformaldehyde (PFA) for 15 minutes at room temperature and treated with RNase (20 mg/ml in 2×SSC) for 30 minutes at 37°C. 100 ng of labeled and heat denatured probe (in 50% deionized formamide, 10% dextran sulphate, 1×SSC and 250 mg/ml herring sperm DNA) were added to parasite preparations before hybridization at 95°C for 3 minutes and at 37°C for 12 hours. After hybridization, parasites were washed twice in 50% deionized formamide/2×SSC at 45°C, twice in 2×SSC first at 45°C then 50°C and once in 4×SSC at room temperature. Blocking with 1% BSA in PBS was followed by incubation with Avidin-Rhodamine (Roche Applied Science) in 1% BSA in PBS for detection of the biotinylated Rep20 probes. Parasites were finally washed once in 1% BSA in PBS and twice in a solution of 100 mM Tris-HCl/150 mM NaCl/0.5% (v/v) Tween 20 at room temperature and mounted in Vectashield (Vector Laboratories). Preparations were visualized using a Leica DMRE microscope and imaged with a Hamamatsu C4880 cooled CCD camera.

### Multiplication rate determination

All parasite clones were initially synchronized with 5% D-sorbitol treatment. Each clone was diluted to a low level of parasitemia (geomean = 0.3%) at schizont stage and thin blood smears were prepared. Cultures were subsequently incubated for 12–16 hours to allow invasion of RBCs and blood smears of the ring-stage parasites were prepared. The smears were stained with Giemsa and the number of IEs with schizonts (parasitemia_start_) or rings (parasitemia_new_) were assessed visually by light microscopy. A minimum of 1000 IEs were counted per smear (geomean = 1500). Data from a minimum of three biological replicates were generated for each clone. The multiplication rate for each clone was obtained by calculating the fold change in parasitemia (parasitemia_new_/parasitemia_start_) for each 48 hour cycle.

### Relative estimation of transcript versus protein abundance

The plot in Figure 5D was generated using the quantitative real-time PCR data to calculate the relative transcript copy number of the dominant transcripts in each parasite clone. Protein expression levels of the corresponding transcripts were assessed using a semi-quantitative approach based on measurements of the signal intensities of the PfEMP1 bands on western blots. Relative quantity (RQ) was obtained by dividing the target quantity with the quantity of the endogenous control in each sample. Seryl t-RNA synthetase and Pfhsp70 were used as endogenous controls for transcript versus protein data respectively.

## Supporting Information

Figure S1 (0.54 MB PNG)

Table S1 (0.01 MB PDF)
