# Supplementary figures and images for: Default Pathway of var2csa Switching and Translational Repression in Plasmodium falciparum
Source: PLoS One. 2008 Apr 23;3(4):e1982. doi: 10.1371/journal.pone.0001982 (PMC2292259; doi:10.1371/journal.pone.0001982)

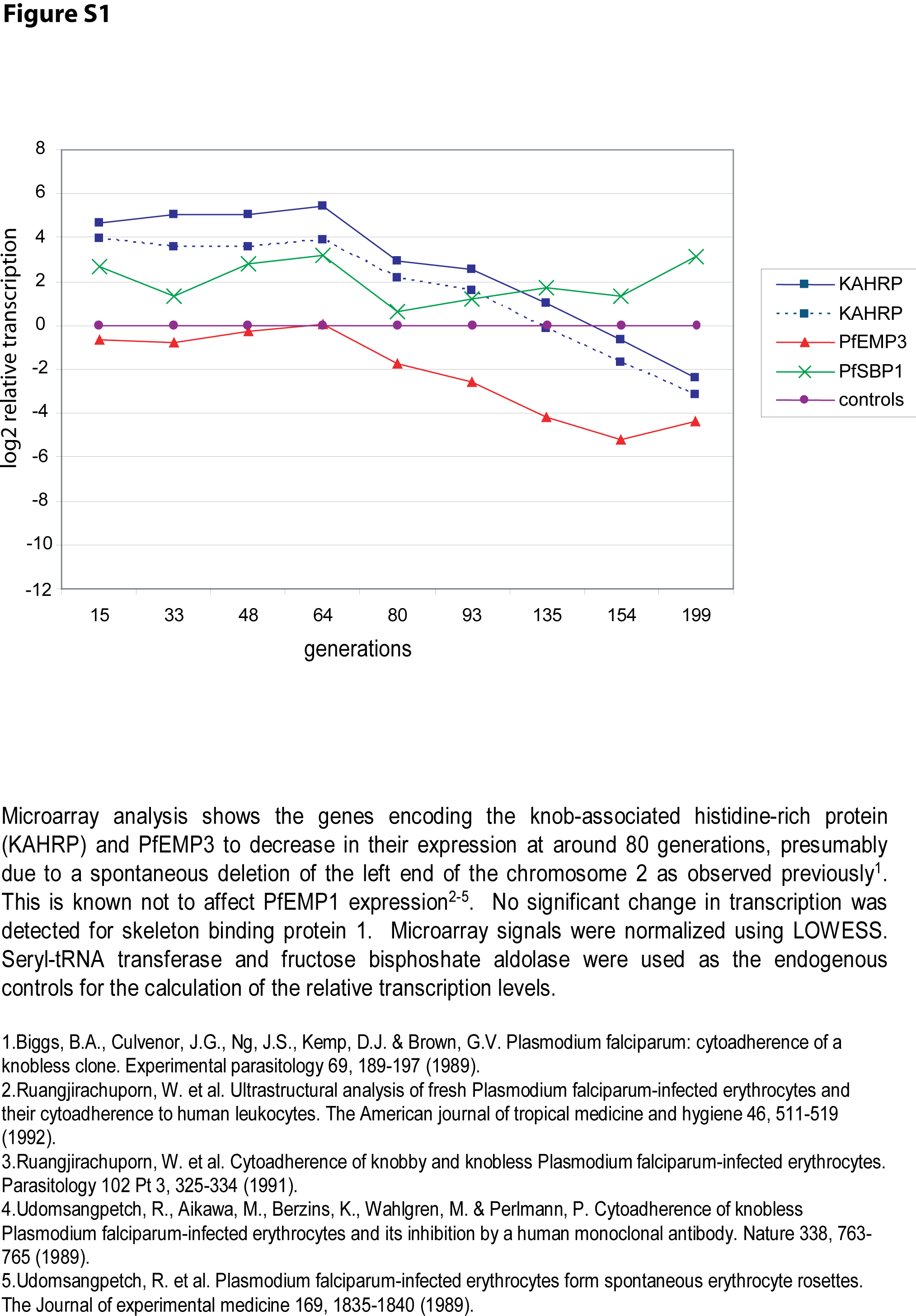

Supplement: Figure S1 — (0.54 MB PNG) [file pone.0001982.s001.png]
