# Supplementary material for: Default Pathway of var2csa Switching and Translational Repression in Plasmodium falciparum
Source: PLoS One. 2008 Apr 23;3(4):e1982. doi: 10.1371/journal.pone.0001982 (PMC2292259; doi:10.1371/journal.pone.0001982)
Supplement: Table S1 — (0.01 MB PDF) [file pone.0001982.s002.pdf]

**Table S1a**. Genes downregulated at 33 generations and 199 generations (vs. 15 generations) observed in microarray studies.

| PlasmoID | Annotation | 15 generations | 33 generations | fold-change | M-value | p-value |
|---|---|---|---|---|---|---|
| PF10_0350 | hypothetical protein | 3125 | 12625 | 0.25 | -2.01 | 3.16E-06 |
| PF11_0503 | hypothetical protein | 762.1 | 2372 | 0.32 | -1.64 | 1.85E-05 |
| PFI1735c | hypothetical protein | 263.5 | 728.8 | 0.36 | -1.47 | 8.11E-05 |
| PFD0630c | PfEMP1 (PFD0630c) | 795.4 | 2081 | 0.38 | -1.39 | 5.47E-07 |
| PFD0630c | PfEMP1 (PFD0630c) | 1641 | 3900 | 0.42 | -1.24 | 0.00018 |
| PFD0630c | PfEMP1 (PFD0630c) | 3401 | 7622 | 0.45 | -1.16 | 6.02E-07 |
| PFI0110c | protein kinase | 262.4 | 686.2 | 0.38 | -1.39 | 0.00027 |
| PFI1755c | hypothetical protein | 263.5 | 678 | 0.39 | -1.36 | 0.000694 |
| PF08_0103 | PfEMP1 (PF08_0103) | 432.6 | 1077 | 0.40 | -1.32 | 0.000177 |
| PF08_0103 | PfEMP1 (PF08_0103) | 264 | 564.5 | 0.47 | -1.10 | 0.000683 |
| PFD1185w | hypothetical protein | 245.8 | 548.8 | 0.45 | -1.16 | 0.000267 |
| PFA0620c | glutamate acid rich protein | 669.1 | 1449 | 0.46 | -1.11 | 0.000405 |
| MAL7P1.172 | hypothetical protein | 1009 | 2144 | 0.47 | -1.09 | 0.000502 |
| MAL7P1.29 | hypothetical protein | 916.1 | 1899 | 0.48 | -1.05 | 0.000601 |

| PlasmoID | Annotation | 15 generations | 199 generations | fold-change | M-value | p-value |
|---|---|---|---|---|---|---|
| PFB0100c | knob assoicated histidine-rich protein | 60705 | 394 | 0.01 | -7.3 | 0.0242 |
| PFB0100c | knob assoicated histidine-rich protein | 28902 | 212.5 | 0.01 | -7.1 | 0.0242 |
| PFB0100c | knob assoicated histidine-rich protein | 26732 | 235.5 | 0.01 | -6.8 | 0.0242 |
| PFB0100c | knob assoicated histidine-rich protein | 19442 | 179.2 | 0.01 | -6.8 | 0.0242 |
| PFD0630c | PfEMP1 (PFD0630c) | 2795 | 92.75 | 0.03 | -4.9 | 0.0018 |
| PFD0630c | PfEMP1 (PFD0630c) | 1416 | 85.5 | 0.06 | -4.0 | 0.0358 |
| PFD0630c | PfEMP1 (PFD0630c) | 636 | 87.62 | 0.14 | -2.9 | 0.00287 |
| PFB0095c | PfEMP3 | 994.2 | 96.5 | 0.10 | -3.4 | 0.0386 |
| PFD0310w | sexual stage-specific protein precursor | 5777 | 840 | 0.15 | -2.8 | 0.0404 |
| PFD0310w | sexual stage-specific protein precursor | 7637 | 1,136 | 0.15 | -2.7 | 0.0405 |
| PFD0310w | sexual stage-specific protein precursor | 4554 | 691.5 | 0.15 | -2.7 | 0.0405 |
| PF13_0011 | *P. falciparum* gamete antigen 27/25 | 592.2 | 131 | 0.22 | -2.2 | 0.0421 |
| PFL0795c | hypothetical protein | 409.7 | 93 | 0.23 | -2.1 | 0.0424 |
| PF10_0374 | Pf 11-1 protein | 469.3 | 113.8 | 0.24 | -2.0 | 0.0428 |
| PF08_0103 | PfEMP1 (PF08_0103) | 356.2 | 98.75 | 0.28 | -1.9 | 0.0439 |
| PF10_0164 | hypothetical protein | 636.8 | 180.5 | 0.28 | -1.8 | 0.0443 |
| PFI1735c | hypothetical protein | 274.5 | 90.5 | 0.33 | -1.6 | 0.0473 |
| PF14_0010 | glycophorin binding protein-related antigen | 377.9 | 125.2 | 0.33 | -1.6 | 0.0466 |
| PF10_0350 | hypothetical protein | 5780 | 2,008 | 0.35 | -1.5 | 0.0477 |
| PFI1755c | hypothetical protein | 258.2 | 91.75 | 0.36 | -1.5 | 0.0481 |

**Table S1b**. Genes upregulated at 33 generations and 199 generations (vs. 15 generations) observed in microarray studies.

| PlasmoID | Annotation | 15 generations | 33 generations | fold-change | M-value | p-value |
|---|---|---|---|---|---|---|
| PFL0030c | PfEMP1 (var2csa) | 255.9 | 691.2 | 2.70 | 1.4 | 5.63E-05 |
| PFL0030c | PfEMP1 (var2csa) | 255.4 | 726.8 | 2.85 | 1.5 | 0.00042 |
| PFL0030c | PfEMP1 (var2csa) | 253.6 | 712.2 | 2.81 | 1.5 | 1.10E-05 |
| PF10_0212 | hypothetical protein | 978.3 | 2544 | 2.60 | 1.4 | 0.000572 |
| PFB0365w | hypothetical protein | 529.6 | 1199 | 2.26 | 1.2 | 0.000295 |
| PFI0160w | hypothetical protein | 443.9 | 972.8 | 2.19 | 1.1 | 8.28E-05 |

| PlasmoID | Annotation | 15 generations | 199 generations | fold-change | M-value | p-value |
|---|---|---|---|---|---|---|
| PFL0030c uORF | PfEMP1 (var2csa) | 166.7 | 1974 | 11.8 | 3.6 | 2.42E-07 |
| PFL0030c | PfEMP1 (var2csa) | 191.2 | 4618 | 24.2 | 4.6 | 1.03E-09 |
| PFL0030c | PfEMP1 (var2csa) | 222.6 | 9091 | 40.8 | 5.4 | 4.71E-08 |
| PFL0030c | PfEMP1 (var2csa) | 264.8 | 7780 | 29.4 | 4.9 | 3.03E-09 |
| PFL0030c | PfEMP1 (var2csa) | 228.3 | 8415 | 36.9 | 5.2 | 4.23E-08 |
| PFL0030c | PfEMP1 (var2csa) | 237.4 | 10018 | 42.2 | 5.4 | 1.11E-08 |
| PFE1615c | hypothetical protein | 120.7 | 301 | 2.5 | 1.3 | 0.000145 |
| PF11_0040 | early transcribed membrane protein 11.2 | 8920 | 18290 | 2.1 | 1.0 | 0.00131 |
